# Protein crystallization and structure determination at room temperature in the CrystalChip

**DOI:** 10.1002/2211-5463.13932

**Published:** 2024-11-21

**Authors:** Petr Pachl, Léna Coudray, Romain Vincent, Léa Nilles, Hélène Scheer, Christophe Ritzenthaler, Adéla Fejfarová, Pavlína Řezáčová, Sylvain Engilberge, Claude Sauter

**Affiliations:** ^1^ CNRS, Architecture et Réactivité de l'ARN, UPR 9002, Institut de Biologie Moléculaire et Cellulaire Université de Strasbourg France; ^2^ Institute of Organic Chemistry and Biochemistry Czech Academy of Sciences Praha 6 Czech Republic; ^3^ CNRS, Institut de Biologie Moléculaire des plantes, UPR 2357 Université de Strasbourg France; ^4^ CNRS, CEA, Institut de Biologie Structurale (IBS) Université Grenoble Alpes France

**Keywords:** CrystalChip, crystallization, microcrystals, microfluidics, serial crystallography

## Abstract

The production of high‐quality crystals is a key step in crystallography in general, but control of crystallization conditions is even more crucial in serial crystallography, which requires sets of crystals homogeneous in size and diffraction properties. This protocol describes the implementation of a simple and user‐friendly microfluidic device that allows both the production of crystals by the counter‐diffusion method and their *in situ* analysis by serial crystallography. As an illustration, the whole procedure is used to determine the crystal structure of three proteins from data collected at room temperature at a synchrotron radiation source.

## Graphical abstract

This protocol describes the implementation of a simple and user‐friendly microfluidic device called CrystalChip that allows the production of crystals by the counter‐diffusion method and their *in situ* analysis by serial crystallography. As an illustration, it is applied to determine the 3D structure of three proteins from data collected at room temperature at a synchrotron radiation source.

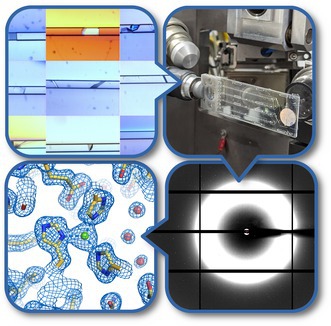

**Abbreviations**
DLS: dynamic light scattering
HEWL: hen egg white lysozyme
PEG: polyethylene glycol
XFEL: X‐ray free‐electron laser
XRD: X‐ray diffraction

Over the last decade, the emergence of intense X‐ray sources such as X‐ray free‐electron lasers (XFELs) and 4th generation synchrotrons led to the development of serial crystallography [1, 2, 3]. Unlike traditional data collection performed on single crystal rotating in the X‐ray beam at cryogenic temperature, a series of randomly oriented micro‐ or nano‐crystals are analyzed at room temperature to complete a full dataset. At XFEL, hundreds of thousands of crystals are sequentially hit by a highly energetic beam, each of them producing a single image before being destroyed, according to the ‘diffraction before destruction’ principle. Diffraction patterns from the resulting series of still images are combined to reconstitute a complete dataset and solve the 3D structure. The same strategy was adapted to characterize micron‐sized crystals at synchrotrons. However, because a synchrotron beam is less intense by several orders of magnitude, it is generally possible to measure a partial dataset on each crystal by collecting small rotation wedges before radiation damage causes the resolution to drop. This dramatically reduces from thousands to a few dozen the number of samples required to determine a structure [4].

Also, with both types of X‐ray sources, serial crystallography has the advantage of operating at more physiological temperatures and allows real‐time monitoring of dynamic processes within crystals, such as enzymatic catalysis, opening a new field of investigation in structural biology with the possibility of producing real molecular films [5, 6, 7, 8, 9].

In serial crystallography as in single‐crystal crystallography, the limiting step is the production of crystals [10, 11]. Good control of crystallization conditions and their reproducibility constitute an even more crucial issue in serial crystallography, because it requires series of crystals that are homogeneous in size and quality, while dealing with limited quantities of biomolecules. From this point of view, microfluidic technologies appeared very promising since their introduction in the early 2000s in the field of biocrystallogenesis [12, 13], because they allow an extreme miniaturization and high‐throughput implementations of crystallization experiments, while providing an ideal convection‐free environment for the growth of quality crystals [14]. It is also an efficient way to bring crystals into the X‐ray beam for serial diffraction experiments [15, 16].

This article presents a pipeline for structure determination based on serial crystallography and exploiting a new microfluidic device (Fig. 1) that miniaturizes an efficient crystallization method and allows the direct *in situ* characterization of resulting crystals by XRD. Microcrystals are produced by the method of counter‐diffusion [17] that exploits the convection‐free environment in the chip to create a gradient of supersaturation while the crystallization agent or crystallant diffuses along the microchip channels [18, 19], thus favoring an optimal screening of nucleation and growth conditions. As soon as crystals appear, they can be characterized at room temperature within the chip, that is, without requiring potentially deleterious manipulation, and can reveal their full diffraction potential, often characterized by a very low mosaicity. Partial datasets resulting from small‐wedged data collection on a series of crystals are compared, sorted, and then combined to lead to the determination of the 3D structure of the crystallized biomolecule. As an illustration, the protocol was applied to the structure determination of three proteins—hen egg white lysozyme (HEWL) as model enzyme, a human enzyme and a protein from plant—and is detailed step by step from crystallization to the final crystal structure.

**Fig. 1:**
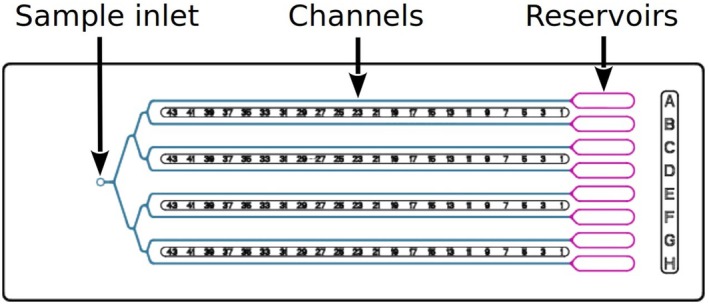
The CrystalChip has the size of a standard microscope slide (7.5 cm × 2.5 cm) and contains eight crystallization channels (4.5 cm × 80 μm × 80 μm). The sample is loaded with a regular P10 pipette in the left inlet and distributed in the channels through a tree‐like connection (colored in blue). Crystallization solutions are deposited in the reservoirs (colored in purple) on the right‐hand side.

## Materials

### Proteins and chemicals

Hen egg white lysozyme was purchased from Sigma‐Aldrich (St. Louis, MO, USA; Fluka Cat. No. 62970‐5G‐F) and solubilized in 50 mm Na‐acetate pH 4.5 at a concentration of 80 mg·mL^−1^. Human carbonic anhydrase II was purified as described in Ref. [20] and stored at 25 mg·mL^−1^ in 50 mm Tris/HCl pH 7.5. Before crystallization, the protein sample was concentrated to 35 mg·mL^−1^ via centrifugal ultra‐filtration using Amicon® Ultra Centrifugal Filter (10 kDa MWCO; Millipore, USA). RNase Three‐like 4 (RTL4) from *Arabidopsis thaliana* was expressed in *Escherichia coli*, purified to homogeneity by a combination of affinity and size exclusion chromatography, and concentrated to 4 mg·mL^−1^ in 50 mm HEPES‐Na pH 7.5, 50 mm NaCl (Hélène Scheer (HS) et al., in preparation). Chemicals used to prepare crystallization solutions were purchased from Sigma‐Aldrich and Hampton Research (Aliso Viejo, CA, USA).

### Microfluidic chips

**Fig. 2:**
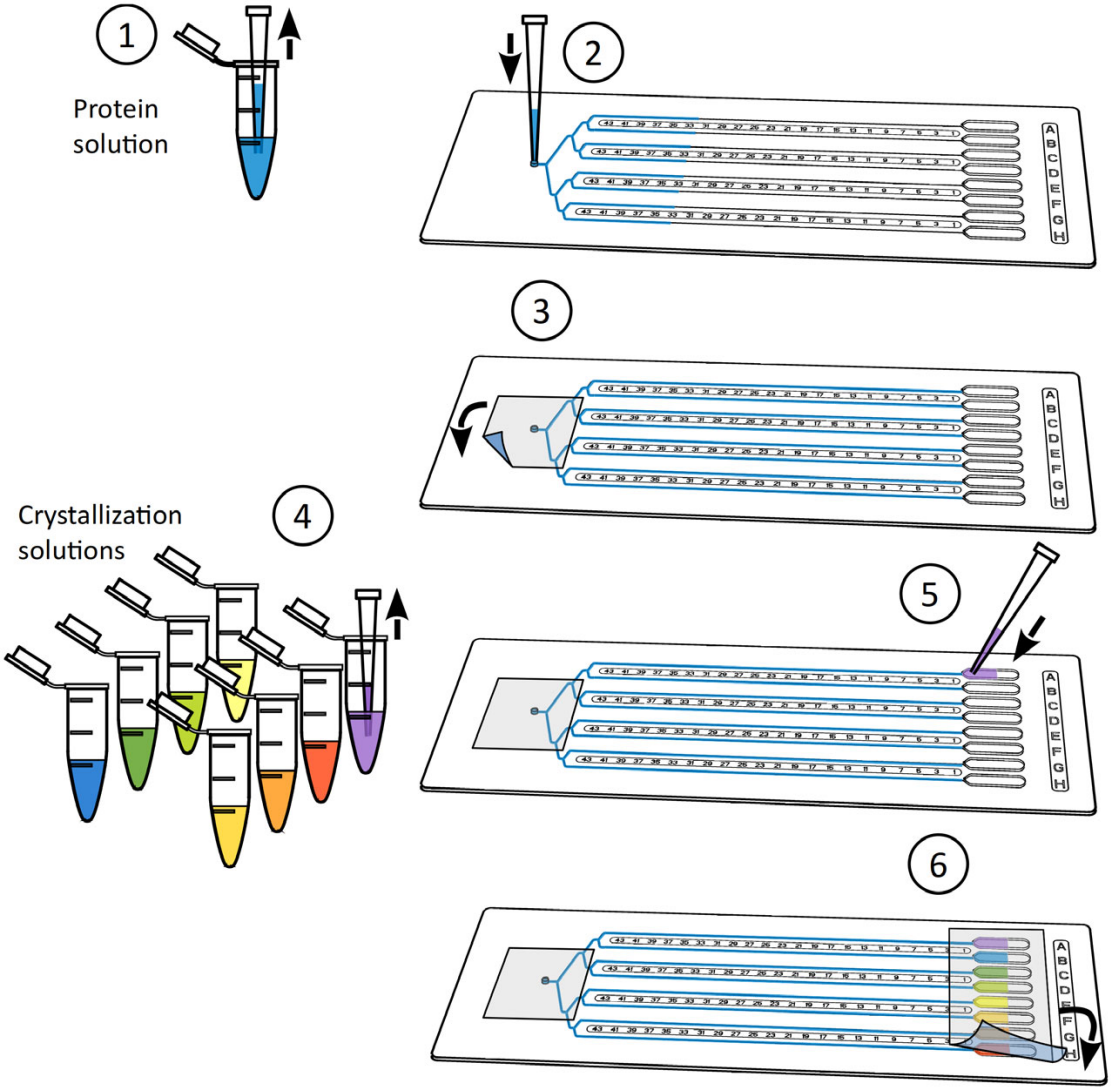
Six steps to set up crystallization assays in CrystalChip.

CrystalChips are designed to perform counter‐diffusion based crystallization experiments in eight microfluidic channels (Fig. 1). Their geometry and material were also optimized to facilitate *in situ* crystal characterization by X‐ray diffraction [19, 21]. CrystalChips were purchased from Idylle, Paris (www.idylle‐labs.com/crystalchip). The chips are loaded with a regular P10 micropipette and adapted pipette tips are provided with the chips (Fig. 2). Sealing tape such Crystal Clear Sealing Tape (Hampton Research) is used to close the sample inlet and the reservoirs of the chip.

### Chip holders and container

Chip holders were designed to store the chips and place them into the X‐ray beam for diffraction analysis. A light and compact holder [21] was designed to fit the CrystalChip on a standard magnetic goniometer head and is provided with the chip. Two additional holders of SBS microplate format were designed to host 2 or 4 chips (see Fig. 4). At synchrotron beamlines, they can be handled by robotics arms and based on the holder place the chips in the X‐ray beam horizontally or vertically for higher variability during data collection. These SBS holders can be fit into an insulated container for traveling or shipping the chips to the synchrotron source. STL files for 3D printing of the SBS devices and the container are provided as Supporting Information. All three holders can be easily printed with any 3D printer. For printing, we recommend polylactic acid (PLA) or polyethylene terephthalate glycol (PETG) with at least 30% infill.

## Methods

### Setting up crystallization assays

0 Unpack a new chip and check that your P10 pipette tips perfectly fit the sample inlet of the chip to get a good seal.
1 Pipette 5–6 μL of protein solution and insert the pipette tip in the sample inlet perpendicularly to the chip surface (Fig. 2).
2 Gently inject the protein solution and fill the eight channels.
3 The sample solution must reach the end of each channel up to the funnel of the reservoirs.
4 Gently remove the tip and seal the sample inlet with a piece of tape.
5 Deposit 5 μL of crystallization solution in each chip reservoir. The pipette tip must be placed in the funnel toward the channel to avoid the formation of an air bubble between the crystallization and protein solutions.
6 Seal the eight reservoirs with a piece of tape.
7 Annotate your experiment on the flat surface on the left‐hand site of the chip with a permanent marker.

### Chip storage and transportation

**Fig. 3:**
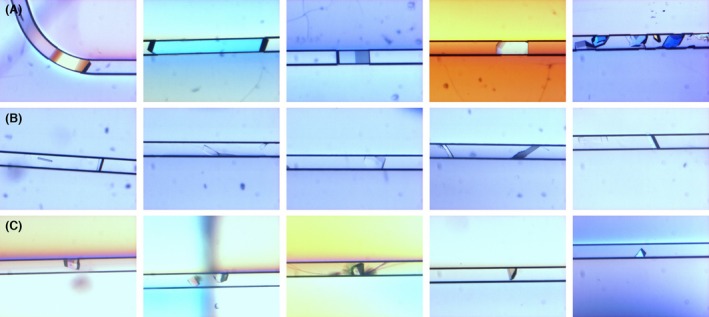
Examples of crystals grown at 293 K in CrystalChip. (A) HEWL crystals grown with a protein solution at 80 mg·mL^−1^ and a crystallization solution containing either 2 m NaCl, 50 mm Na‐acetate pH 4.5 or 1 m NaCl, 10% (w/v) PEG 3350, 50 mm Na‐acetate pH 4.5. (B) Crystals of carbonic anhydrase II grown with a protein solution at 35 mg·mL^−1^ and a crystallization solution containing 50 mm Tris/HCl, 1.6 m sodium citrate, pH 7.8. (C) RTL4 crystals grown with a protein solution at 4 mg·mL^−1^ and a crystallization solution containing 14% (w/v) PEG 3350, 50 mm Bis‐Tris/HCl pH 6.5–7.0. Crystals can grow until they completely fill the channels (section 80 μm × 80 μm).

**Fig. 4:**
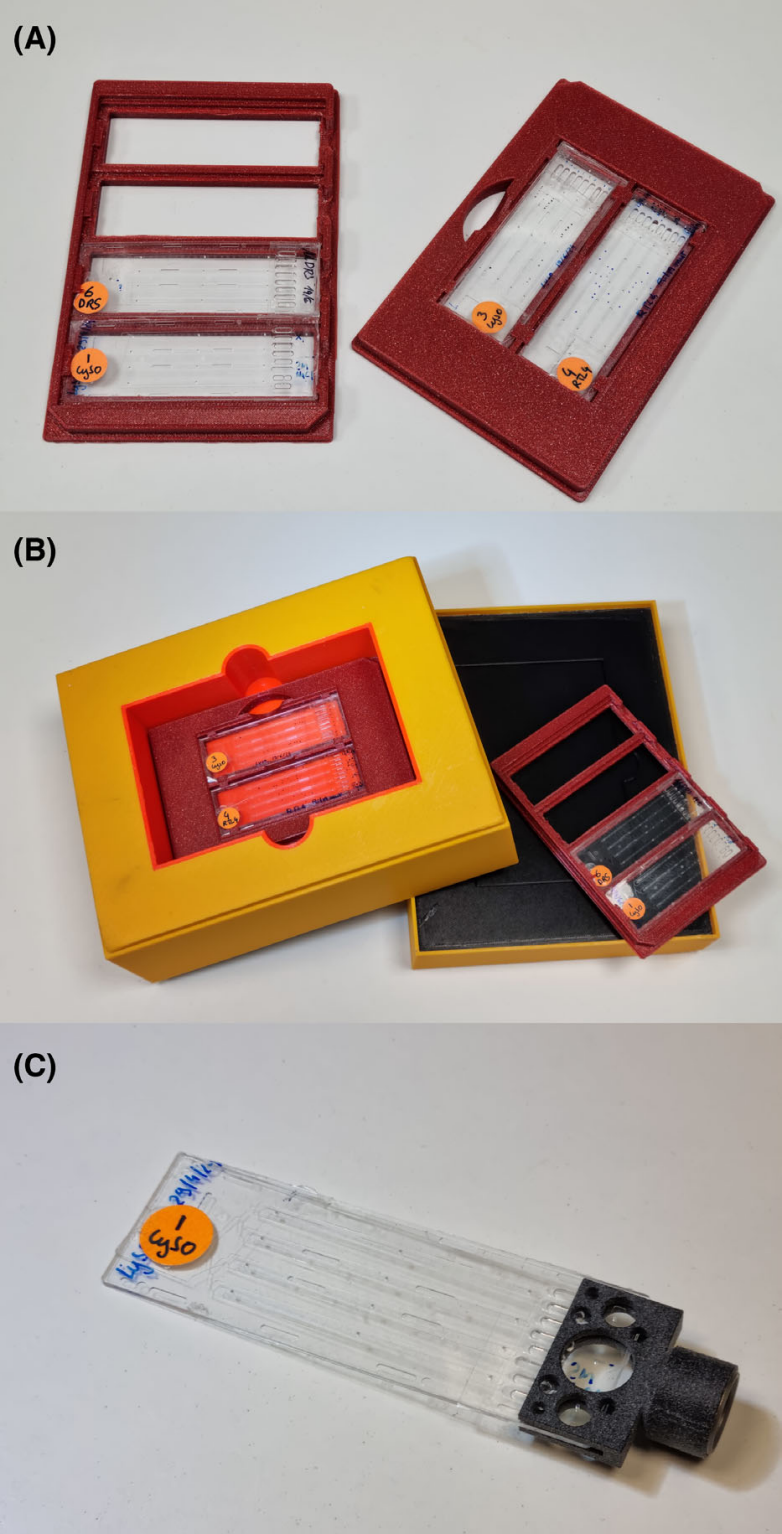
(A) Chips can be clipped on SBS holders for storage at various temperatures. This type of holder can be gripped by robotic arms for data collection at synchrotron beamlines. (B) Chips can be safely carried or shipped to synchrotron facilities using an insulated container. (C) A light holder is used to attach the chip to standard magnetic goniometers (see also Fig. 5).

1. Clip the chips on dedicated holders and incubate them at an appropriate temperature (typically 278 or 293 K).
2. Check the chips daily under the microscope to detect the growth of crystals (Fig. 3).
3. Holders (Fig. 4B) can be safely stacked on top of each other to save space in the incubators or on the shelves.
4. These holders are also very convenient for crystal observation under the microscope or using an imaging system adapted to SBS microplates.
5. To travel with the chips or to send them by express mail to a synchrotron facility, place them with their holder in the insulated container (Fig. 4C). The latter may be additionally packed in a polystyrene box for a better protection against temperature variations.

### *In situ* crystal analysis

**Fig. 5:**
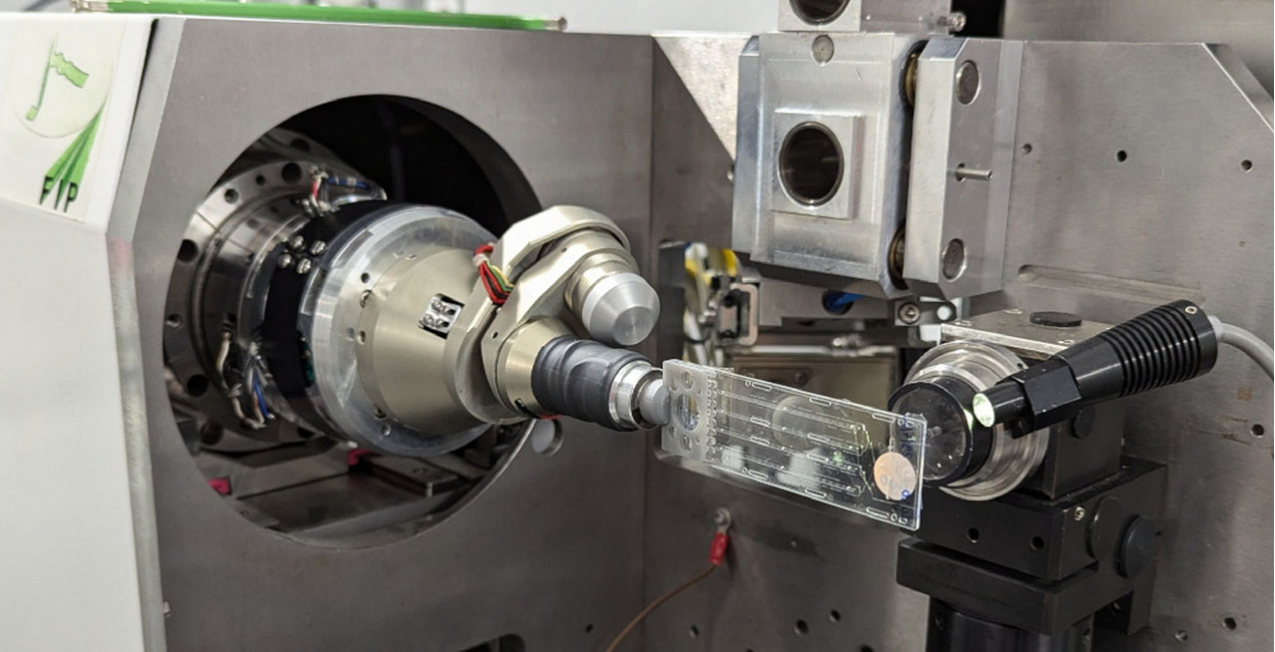
CrystalChip with its holder on the goniometer of beamline BM07‐FIP2 at ESRF.

**Fig. 6:**
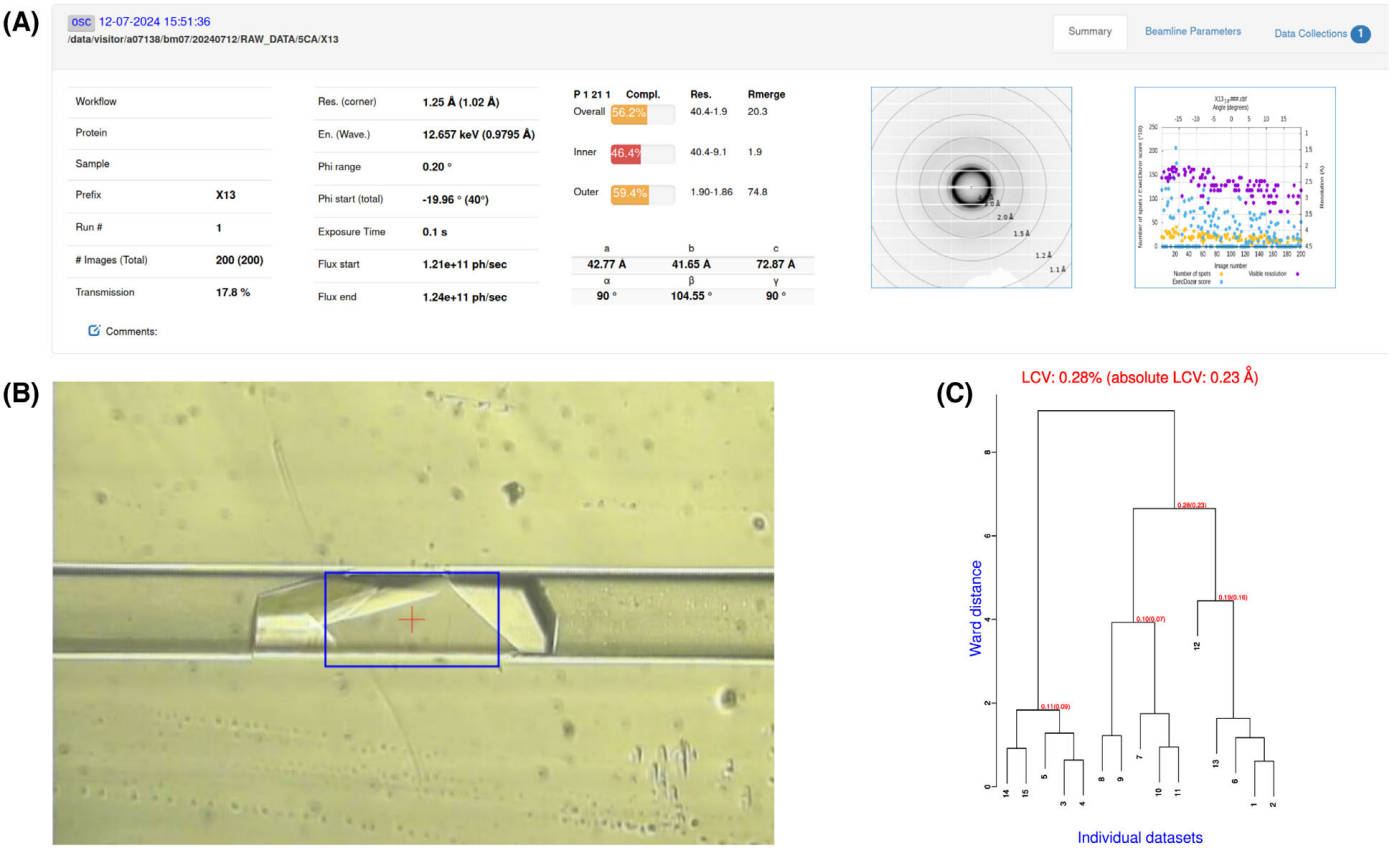
Example of ISPyB interface at ESRF [23] for on‐the‐fly data processing (A) for a monoclinic crystal of carbonic anhydrase II. This automatic pipeline allows a rapid evaluation and an initial sorting of the partial datasets. (B) Crystal centered in the beam symbolized by the blue rectangle (size 200 × 100 μm at beamline BM07‐FIP2). (C) BLEND dendrogram showing the clustering of carbonic anhydrase II datasets as a function of cell parameter similarity. Closest data can then be merged. The variation of cell parameters is very low at room temperature, as low as ~ 0.28% at max in this example.

1. Insert the chip in the light holder and attach the latter to the magnetic head of the goniometer (Fig. 5).
2. Orient the chip vertically, that is, perpendicularly to the X‐ray beam and the on‐axis camera view, with its thick layer on the beam side and its thin layer on the detector side. Letters and numbers must be readable with the alignment camera.
3. Translate the chip to center the beam on a crystal in the top channel (Fig. 6A).
4. Attenuate the X‐ray beam to 5–20% transmission to avoid rapid decay due to radiation damage at room temperature (adapt with beamline scientists).
5. Collect a dataset with a rotation from −15° to +15° with respect to the vertical position.
6. Launch automatic data processing to evaluate the diffraction quality (Fig. 6B).
7. Translate the chip along the channel to center on the next crystal in the channel. When reaching the end of a channel, translate the chip to explore the parallel channel.
8. Repeat these operations to characterize all crystals of the chip (serial strategy).
9. In case the goniometer has limited translation possibilities, the chip can be flipped by 180° on the holder to reach its second half.

### Data processing and structure determination

**Fig. 7:**
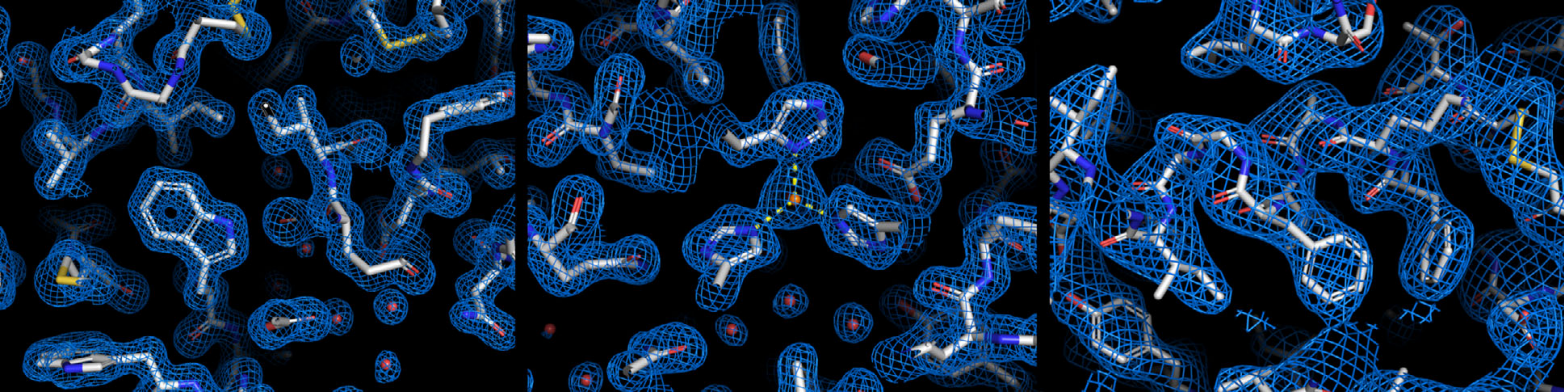
Crystal structures of HEWL (left), carbonic anhydrase II (middle), and RTL4 (right) in electron density maps (contoured at 1.2 sigma) refined at 1.5, 1.8, and 2.8 Å, respectively, from crystals grown in CrystalChips. The figure was prepared with pymol 3.10.14 (Schrödinger, LLC, New York, USA).

1. Automatic data processing [22, 23] should be launched during data collection to get a first evaluation of crystal diffraction quality (Fig. 6B).
2. Select best samples based on this first evaluation and reprocess each individual dataset with the xds package [24].
3. As small crystals may be misaligned with the beam at the beginning or at the end of the rotation, remove corresponding low‐dose images.
4. Compare the resulting datasets with the program BLEND from the ccp4 suite [25, 26] and check their isomorphism (i.e., compatibility of their cell parameters).
5. Select clusters of most isomorphous datasets (dendrogram Fig. 6C) and average them with BLEND to obtain a complete dataset in MTZ format (see statistics in Table 1).
6. Alternatively, calculate the average cell parameters of selected datasets with CELLPARM, merge them with xscale and convert the final reflection file in MTZ format using the xds package [24]. All these operations can be conveniently performed with the XDSGUI interface [27].
7. Prepare a molecular replacement model with AlphaFold [28] or from a previously determined structure.
8. Search for a MR solution with phaser [29].
9. Refine the model (Fig. 7) following usual protocols with phenix_refine [30].

**Table 1:** Data collection and refinement statistics.

Protein	Lysozyme	Carbonic anhydrase II	RTL4
Molecular mass (kDa)	14.3	28.8	35.2
Source	*Gallus gallus*	*Homo sapiens*	*Arabidopsis thaliana*
Synchrotron beamline	BM07‐FIP2	BM07‐FIP2	BM07‐FIP2
Temperature (K)	298	298	298
Wavelength (Å)	0.9795	0.9795	0.9795
Distance (mm)	202.8–269.0	202.8–269.0	229.9–391.1
Oscillation (deg/s)	0.20/0.1	0.20/0.1	0.20/0.1
No. of analyzed crystals	15	15	9
No. of merged crystals	7	12	7
Space group	*P*4_3_2_1_2	*P*2_1_	*C*222_1_
*a* (Å)	79.16	42.76	48.13
*b* (Å)	79.16	41.65	101.53
*c* (Å)	38.15	72.87	134.03
Beta (deg)	90.0	104.6	90.0
Asymmetric unit content	1 Monomer	1 Monomer	1 Dimer
Solvent content (%)	41.2	43.6	47.1
Mosaicity (°)	0.045 (±0.010)	0.039 (±0.001)	0.082 (±0.030)
Resolution range (Å)	1.50–35	1.80–50	2.80–50
No. of observed reflections	367 074	160 567	64 222
No. of unique reflections	37 004	44 097	7803
Completeness (%)	99.6	97.7	92.5
*R*_merge_, *R*_meas_ (%)	17.7, 18.6	27.7, 32.2	40.7, 43.4
*I*/sig(*I*)	9.49	4.04	4.40
Redundancy	9.92	3.64	8.23
CC1/2 (%)	99.7	95.8	97.4
High‐resolution shell (Å)	1.50–1.54	1.80–1.85	2.80–2.87
No. of observed reflections	21 510	11 315	3046
No. of unique reflections	2731	3186	441
Completeness (%)	97.9	96.7	74.4
*R*_merge_, *R*_meas_ (%)	219.8, 234.4	152.6, 177.7	212.8, 227.6
*I*/sig(*I*)	0.94	1.18	0.78
Redundancy	7.88	3.55	6.91
CC1/2 (%)	39.5	30.8	30.3
No. of reflections in working/test sets	19 912/996	23 244/1163	7718/382
Final *R*_work_/*R*_free_ (%)	0.138/0.177	0.168/0.215	0.214/0.256
No. of non‐H atoms: protein/solvent	1008/95	2057/205	2131/–
R.m.s. deviations for bonds (Å)/angles (°)	0.004/0.713	0.007/1.092	0.001/0.314
Average *B* factors (Å^2^): overall/protein/solvent	26.8/25.6/40.5	20.4/19.2/32.2	59.3/59.3/–
Ramachandran plot: residues in most favored (%)/allowed (%) regions	98.4/100	96.9/100	96.0/99.6
PDBid	9H3E	9H3H	9H4A

## Tips & Tricks

### Sample loading

- After injecting the protein solution in the sample inlet, 1–2 μL of paraffin oil may be injected to separate the channels from each other. This prevents cross‐contamination if different crystallization solutions are used in the reservoirs.
- After filling the channels with the protein solution, a gel plug (1 μL of 0.5% w/v low gelling point agarose solution) may be formed before depositing the crystallant solution in the funnel of the reservoir to stabilize the interface between the protein and the crystallant solutions.
- To completely avoid microcrystal movements in the channels during transportation and data collection, 0.2–0.3% w/v low gelling point agarose may be incorporated to the protein solution [31, 32, 33].
- Seeding can be easily performed in CrystalChip by adding seeds to the protein solution just before filling the channels to ensure the reproducibility of crystal production [21, 34].

### Crystallization conditions

- When reproducing in CrystalChip crystallization conditions observed in vapor diffusion or batch, increase 1.5–2× the crystallant concentration in the reservoir.
- When performing initial screening for crystallization conditions in CrystalChip, select screen solutions specifically adapted to counter‐diffusion, that is, including high crystallant concentrations to maximize the concentration and supersaturation gradient along the channels [17, 35].
- Protein ligands, enzyme substrates, compounds for fragment screening, and anomalous scatterers for phasing can be added to the reservoir solutions, from the beginning or after crystal growth. They will diffuse in the channels to be incorporated in the crystals [21, 36].

### Crystal observation

- Microcrystals can be identified in CrystalChip using the intrinsic tryptophan fluorescence in proteins under UV illumination [21, 37].
- Microcrystals can also be identified in CrystalChip using the method of trace fluorescence labeling which consists in labeling the protein with a fluorescent probe before crystallization [38, 39].

### Additional possibilities

- CrystalChip is particularly adapted to the crystallization of oxygen‐sensitive samples (e.g., redox enzymes) and can be easily setup in a glove box under inert atmosphere.
- A batch experiment can be prepared by mixing the protein and crystallant solutions, then be loaded into a chip to distribute the crystals in the channels for analysis by XRD.
- CrystalChip is not restricted to the crystallization of biological macromolecules but can also be used with small molecules by playing with solvent—counter‐solvent systems.

## Discussion

The CrystalChip is a new tool in the biochemist's portfolio designed to facilitate access to serial approaches. The three proteins used as illustrations in this work reveal how microfluidics can help the production of quality crystals. Their analysis within the chip itself, without direct manipulation or cryocooling, guarantees to preserve their diffraction properties, as evidenced by the low mosaicity of the collected datasets, typically < 0.1° with a low standard deviation (see Table 1). Serial wedged data collection at room temperature is also compatible with crystals of low symmetry as illustrated by the case of carbonic anhydrase II that crystallizes in monoclinic space group. The number of crystals required to collect a complete dataset will depend on crystal properties (size, solvent content, diffraction quality, symmetry) and the protein sensitivity to radiation damage. Hence, the size of the series will be adapted according to the sample, but from the cases described here and in other studies [4, 21] the analysis of 10–30° wedges on 10–20 crystals is often sufficient. As a large number of (micro)crystals can easily be grown in the channels of the CrystalChip—the total number of crystal per chip was ~ 250, 70, and 40 with HEWL, hCA, and RTL4, respectively—it is possible to extend the series and collect very small‐wedges or even still images with radiation sensitive samples. The compact format of the chip ensures easy transport or even easy shipping by mail, which was the case for the three samples described in the article. The next step in a near future will be the full integration of the CrystalChip on a synchrotron microfocus beamline with the implementation of a fully automatic chip screening pipeline to detect crystals, collect data on the most promising samples, and produce a set of averaged reflections, thus opening the field of serial crystallography to all.

## Conflict of interest

CS designed the chip described in this protocol and codeveloped with Idylle, Paris. However, the work was carried out independently from the company.

## Author contributions

PP and CS conceived and designed the project. PP, LC, RV, AF, HS, CS, and SE carried out the experiments and acquired the data. LN, SE, and CS analyzed and interpreted the data. PR, CR, and CS supervised the experiments, and CS wrote the manuscript.

## Supporting information


**Data S1.** CrystalChipHolders.zip is an archive containing 3D printing files: CrystalChip_x4_holder.stl, CrystalChip_x2_holder.stl: files to print holders for 2 or 4 CrystalChips, respectively (Fig. 4A). CrystalChip_box_*.stl: files to print the different elements (lid, top, inner, outer, bottom parts) of the shipping container hosting a stack of up to 6 chip holders (Fig. 4B).

## Data Availability

The data that support the findings of this study (original XRD images) are openly available through the ESRF data portal at https://data.esrf.fr/doi/10.15151/ESRF‐ES‐1752158517. The structural data described in this study are openly available in the wwPDB at https://doi.org/10.2210/pdb9H3E/pdb, https://doi.org/10.2210/pdb9H3H/pdb and https://doi.org/10.2210/pdb9H4A/pdb.
